# Epstein-Barr virus induces germinal center light zone chromatin architecture and promotes survival through enhancer looping at the *BCL2A1* locus

**DOI:** 10.1128/mbio.02444-23

**Published:** 2023-12-07

**Authors:** Joanne Dai, Elliott D. SoRelle, Emma Heckenberg, Lingyun Song, Jana M. Cable, Gregory E. Crawford, Micah A. Luftig

**Affiliations:** 1 Department of Molecular Genetics and Microbiology, Center for Virology, Duke University School of Medicine, Durham, North Carolina, USA; 2 Center for Genomic & Computational Biology, Duke University, Durham, North Carolina, USA; 3 Division of Medical Genetics, Department of Pediatrics, Duke University, Durham, North Carolina, USA; Dana-Farber Cancer Institute, Boston, Massachusetts, USA

**Keywords:** B-cell, germinal center, EBV, antiapoptosis, chromatin

## Abstract

Epstein-Barr virus (EBV) is a ubiquitous human virus that promotes B-cell activation and maturation through expression of latency proteins and non-coding RNAs. In this study, we provide evidence that EBV mimics the molecular phenotype of germinal center (GC) B cells. EBV infection of primary human B cells promotes their rapid proliferation and GC dark zone (DZ)-like gene expression profile. Following this transient hyperproliferative period, the activation of NF-κB target genes, including *Bcl2a1* (BFL-1), simulates the transition from the DZ to the T-cell supported light zone (LZ). We previously characterized the regulatory landscape of EBV^+^ B cells at the *Bcl2a1* locus defining a key role for the viral EBV nuclear antigen (EBNA) 3A protein in promoting three-dimensional chromatin architecture correlated with BFL-1 expression. Here, we define the global chromatin accessibility of tonsillar B cells and find that naïve and memory B cells have highly similar accessibility profiles that differ substantially from those of DZ and LZ B cells. Notably, multiple regions within the *Bcl2a1* locus are significantly more accessible in DZ and LZ versus naïve and memory subsets. However, we found that BFL-1 upregulation from DZ to LZ correlates with a significant increase in three-dimensional (3-D) chromatin association between accessible upstream enhancer regions and the BFL-1 transcriptional start site. These elements were critical for BFL-1 expression in lymphoblastoid cell lines (LCLs). Moreover, increased BFL-1 expression in LCLs protected against extrinsic apoptosis. Collectively, these results suggest a conserved mechanism underlying BFL-1 upregulation that promotes survival of both LZ and EBV^+^ immortalized B cells.

**IMPORTANCE:**

Epstein-Barr virus has evolved with its human host leading to an intimate relationship where infection of antibody-producing B cells mimics the process by which these cells normally recognize foreign antigens and become activated. Virtually everyone in the world is infected by adulthood and controls this virus pushing it into life-long latency. However, immune-suppressed individuals are at high risk for EBV+ cancers. Here, we isolated B cells from tonsils and compare the underlying molecular genetic differences between these cells and those infected with EBV. We find similar regulatory mechanism for expression of an important cellular protein that enables B cells to survive in lymphoid tissue. These findings link an underlying relationship at the molecular level between EBV-infected B cells in vitro with normally activated B cells *in vivo*. Our studies also characterize the role of a key viral control mechanism for B cell survival involved in long-term infection.

## INTRODUCTION

The Epstein-Barr virus (EBV) is a ubiquitous gamma-herpesvirus that infects >95% of the global adult population. EBV latent infection is life-long and is established in quiescent memory B cells, presumably by undergoing the germinal center (GC) reaction alongside maturing, uninfected B cells (1). In this model of infection, known as the germinal center model, primary EBV infection is asymptomatic and escapes immune detection by robust T-cell-mediated responses. However, in immune-suppressed individuals, such as those infected with HIV or following organ transplant, unchecked EBV infection can give rise to B-cell lymphomas. Worldwide, nearly 200,000 patients each year are diagnosed with EBV-positive cancer.

EBV-driven oncogenesis is modeled through the *in vitro* infection of human B cells, which leads to their growth transformation into immortalized lymphoblastoid cell lines (LCLs). This process requires the expression of six latent proteins: EBV nuclear antigen 1 (EBNA1), EBNA2, EBNA3A, EBNA3C, EBNA-LP, and latent membrane protein 1 (LMP1). The EBNAs are expressed early after infection and regulate viral and host gene expression by hijacking host transcriptional machinery and transcription factors. EBNA2 induces c-Myc expression that drives rapid hyperproliferation and induction of the DNA damage response that limits EBV-mediated transformation (2). Infected B cells in the early phase (approximately 1 week after initial infection) express the latency IIb program, which is characterized by low LMP1 expression. About 3–5 weeks post-infection, EBV^+^ B cells complete the transformation into LCLs and express the latency III program, which is characterized by full expression of all viral products (3, 4). This includes high levels of LMP1, which mimics an active CD40 receptor that signals constitutively through NF-κB and is required for proliferation and survival (5, 6).

Our laboratory has found that the early and late phases of EBV are transcriptomically distinct (7) and utilize unique strategies to promote apoptosis resistance (8). Apoptosis is regulated by complex protein-protein interactions between pro- and anti-apoptotic members of the BCL2 protein family. While uninfected B cells are dependent upon the anti-apoptotic protein BCL2 for survival, early infected B cells depend upon BCL2 and MCL-1, and late infected B cells at the LCL stage upregulate dependency upon BFL-1. We determined that upregulation of BFL-1, which is expressed from the *BCL2A1* gene, requires EBNA3A, a viral transcriptional cofactor that activates and represses viral and host gene expression by regulating enhancer activity and chromatin architecture (9–12). To promote BFL-1 transcription, EBNA3A promotes the looping of upstream enhancer regions to the transcriptional start site (TSS) (13). In EBNA3A-null LCLs, these enhancer-promoter interactions are lost, and levels of active transcription machinery at the TSS are reduced. In addition to its role in inhibiting expression of the pro-apoptotic protein BIM, EBNA3A promotes survival in EBV-immortalized LCLs through chromatin-mediated regulation of apoptotic proteins.

Like LCLs, germinal center light zone (GC LZ) B cells express high levels of BFL-1 mRNA induced by strong activation of the CD40 receptor (14); however, the non-viral mechanisms underlying BFL-1 upregulation remain unknown. BFL-1 expression is so high that it is frequently used as a marker to distinguish GC LZ B cells from other B lymphocytes (15). Historically, the physiological role of BFL-1 was difficult to study because of gene quadruplication in mice, whereas humans express one gene (16). However, *in vivo* studies made possible by transgenic RNAi mouse models suggest that BFL-1 is important for the survival of activated, mature B cells (17, 18). This has not yet been testable in human GC B cells, which are especially sensitive to spontaneous apoptosis *ex vivo* and are genetically intractable, making it challenging to study BFL-1 upregulation in GC LZ B cells.

In this study, we characterize the chromatin architecture and enhancer-mediated BFL-1 transcription in EBV-immortalized LCLs and find that this architecture strongly resembles that in GC LZ B cells, suggesting that EBV infection *in vitro* intrinsically recapitulates certain aspects of the GC reaction. Because human GC LZ B cells are not amenable to genetic experiments, we use LCLs to perform functional analysis of BFL-1 enhancer-promoter interactions. Our analysis also reveals a large overlap of dynamically expressed gene targets in GC B cells and EBV-infected B cells. By characterizing the chromatin landscape of LCLs and GC LZ B cells, we present data that supports the long-standing germinal center model of EBV infection *in vivo* that postulates a route for virus-infected B cells through the germinal center reaction to establish life-long latent infection.

## MATERIALS AND METHODS

### Cell culture

LCLs used in this study were also used in Price et al. (8). In short, peripheral blood mononuclear cells (PBMCs) were harvested from human donors through the Gulf Coast Regional Blood Center (Houston, TX). CD19^+^ B cells were isolated from PBMCs using the BD iMag Negative Isolation Kit (BD, 558007) and subsequently infected with the wild-type (WT) B95-8 Epstein-Barr virus strain as previously described (19). The recombinant EBNA3A-null virus that was used in this study and in reference (8) was generated in reference 12. LCLs were maintained in RPMI supplemented with 10% fetal bovine serum (FBS) (Corning), 2 mM L-glutamine, 100 U/mL penicillin, and 100 µg/mL streptomycin (Invitrogen).

Tonsillar B cells were isolated from discarded, anonymized tonsillectomies from the Duke Biospecimen Repository and Processing Core (Durham, NC). Tonsil tissue samples were manually disaggregated, filtered through a cell strainer, and isolated by layering over a cushion made from Histopaque-1077 (H8889; Sigma-Aldrich). Harvested lymphocytes were washed three times with fluorescence activated cell sorting (FACS) buffer (5% FBS in phosphate-buffered saline [PBS]) and stained for surface markers using anti-CD19-PE (363003, Biolegend, RRID: AB_2564125), anti-IgD-FITC (348206, Biolegend, RRID: AB_10612567), anti-CD38-APC (303510, Biolegend, RRID: AB_314362), anti-CD83-BV421 (305324, Biolegend, RRID: AB_2561829), and anti-CXCR4-PeCy7 (560669, BD Biosciences, RRID: AB_1727435). For flow cytometry analysis, stained cells were analyzed on a BD FACS Canto II and sorted on a MoFlo Astrios Cell Sorter at the Duke Cancer Institute Flow Cytometry Shared Resource (Durham, NC). Typical yields from cell sorting were 1 × 10^6^ naïve cells, 0.5–1 × 10^6^ DZ/LZ GC B cells, and ~0.1 × 10^6^ plasma cells per tonsil.

### Chromatin immunoprecipitation (ChIP)

ChIP was performed as described previously (12) using an Active Motif ChIP-IT PBMC assay kit (catalog no. 53042). Cells were fixed for 8 min and sonicated for 60 min (30 s on, 30 s off) on high with a standard Diagenode Bioruptor Sonicator. Sonicated ChIP input was reverse crosslinked and purified to run on a 1.5% agarose gel to ascertain appropriate fragment sizes. Antibodies used were rabbit anti-YY1 (Active Motif, 61779, RRID:AB_2793763), rabbit anti-H3K27ac (Active Motif, 39135, RRID:AB_2614979), rabbit anti-H3Kme1 (EMD Millipore, 07-436), rabbit IgG isotype control (Thermo Fisher, 02-6102).

### CUT&RUN assays

CUT&RUN was performed with the CUTANA ChIC/CUT&RUN Kit (Epicypher, 14-1048). A total of 500,000 cells for each condition were fixed with 1% formaldehyde for 1 min, and the protocol for fixed cells was followed as described in the manual. Antibodies used were rabbit IgG (Epicypher, 13-0042), rabbit anti-YY1 (Cell Signaling Technology, 46395S) at 1:50, and rabbit H3K27ac (ActiveMotif, 39034) at 1:50. Libraries were prepared using the CUTANA ChIC/CUT&RUN Library Prep Kit (Epicypher, 14-1001). Samples were run on the Illumina NextSeq 1000 P2 flow cell, with 50-bp paired end reads. Sequenced reads were analyzed using the nf-core/cutandrun pipeline (20, 21). Peak calling was performed using SEACR (22).

### Chromatin conformation capture (3C)

3C was performed as previously described (23). In brief, chromatin was crosslinked with formaldehyde and isolated from LCLs, whereupon it was digested overnight with HindIII-HF (NEB, R3104M). Digested chromatin was then diluted and ligated overnight at 16°C with T4 DNA ligase (NEB, M0202M). Reversal of crosslinks, proteinase K digestion, and phenol-chloroform extraction yielded DNA circles that were assayed by TaqMan quantitative PCR (qPCR). Cycle threshold (Ct) values were normalized to enrichment to a region closest to the anchor primer.

### Assay for transposase-accessible chromatin with sequencing (ATAC-seq)

Three biological replicates of sorted CD19^+^ B cells (naïve, memory, GC DZ, GC LZ, and plasma cells) were isolated and viably frozen. After thawing, 50,000 cells per sample were processed for ATAC-seq as described in reference 24. Samples were run on two lanes of the Illumina 4000 HiSeq. ATAC-seq data were processed as previously described. Adaptor sequences were trimmed and mapped to hg38 using Bowtie2 (25). Reads were then filtered for duplicates. The filtered reads for each sample were merged, and peak calling was performed by MACS2 (26).

### dCas9-KRAB systems

A short-guide RNA targeting the BFL-1 transcriptional start site was cloned into the BsmbI-digested dCas9-KRAB-GFP (Addgene #71237, RRID: Addgene_71237) or lentiguide-puromycin (Addgene #52963, RRID: Addgene_52963) vector. Ligated vector was then transformed into Stbl2 and sequence confirmed with the hU6 primer. One microgram plasmid was then transfected with 1 µg psPAX2 (Addgene #12260, RRID: Addgene_12260) and 100 ng pMD2.G (Addgene #12259, RRID: Addgene_12259) in 293Ts with Mirus LTI reagent in antibiotic-free media and harvested 48 and 72 hours post-transfection. Virus was then filtered and concentrated prior to transducing LCLs. LCLs transduced with dCas9-KRAB-GFP were then sorted for GFP^+^ events and grown out. LCLs transduced with lentiguide-puromycin were selected with 0.8 µg/mL puromycin for 4–7 days and then transduced with TRE-HAGE-dCas9-KRAB lentivirus, which was generated similarly. Transduced cells were then selected with 100 µg/mL G418 in RPMI supplemented with 15% Tet-free FBS. To induce TRE-HAGE-dCas9-KRAB activity, cells were treated with 3 µg/mL doxycycline for 48 hours and then harvested for mRNA. To show Dox-specific effects, treated cells were washed twice with Tet-free media and cultured for 48 hours before harvesting for mRNA.

### Cas9 RNP transfection

Cas9 RNP transfections were performed based on manufacturer’s instructions (Thermo Fisher Scientific, TrueCut Cas9 Protein v2). Single-guide RNAs (sgRNAs) were synthesized by Synthego. In short, Cas9 protein and sgRNAs were mixed in a ratio of 1:3 and incubated in Belzer’s solution for 20 min at room temperature. Cells were used at a final concentration of 10 × 10^6^ /mL. Microporations were performed with the Neon system with 10-µL tips. Cells were recovered in RPMI supplemented with 15% FBS and no antibiotic. Twenty-four hours after electroporation, cells were supplemented with 1% penicillin-streptomycin-glutamine. mRNA and genomic DNA were harvested 5 days post transfection to assay BFL-1 mRNA levels by qPCR and sgRNA-targeted genomic deletions by PCR, which was performed with GoTaq master mix (Promega).

### Quantitative PCR

mRNA was isolated from samples using the Promega SV 96 Total RNA Isolation System and reverse transcribed into cDNA using the High-Capacity cDNA Reverse Transcription Kit from Thermo Fisher. qPCR was performed using the Power SYBR Green PCR Master Mix from Thermo Fisher. Relative mRNA values were calculated using the ΔΔCt method and normalizing to SetDB1 or ALAS1 housekeeping genes. Oligonucleotides used are given in Table 1.

**TABLE 1:** Oligonucleotides

Oligos used for qPCR (5′−3′)		
Oligo name	Forward	Reverse
BFL-1	TTACAGGCTGGCTCAGGACT	AGCACTCTGGACGTTTTGCT
SetDB1	GACTACAATACCGGGACAGTAGC	CCCAGCATCACCTGAATCAAT
BCL6	GGAGTCGAGACATCTTGACTGA	ATGAGGACCGTTTTATGGGCT
Blimp1	TGAAGATGGGAGCGAAGAGAT	ACCTTGCCCTGCTTAACACAA
EBI3	TCATTGCCACGTACAGGCTC	GGGTCGGGCTTGATGATGTG
c-Myc	CTCCATGAGGAGACACCGC	GAGCCTGCCTCTTTTCCACA
BATF	TGCTCAGAGAAGTCGGAAGAA	TGGCACAAAGTTCATAGGGCA
ALAS1	CGCCGCTGCCCATTCTTAT	TCTGTTGGACCTTGGCCTTAG

Oligos used for ChIP (5′−3′)		
Oligo name	Forward	Reverse
Myo	GGAGAAAGAAGGGGAATCACA	GATAAATATAGCCAACGCCACA
CXCL-10-TSS	TCCCTCCCTAATTCTGATTGG	AGCAGAGGGAAATTCCGTAAC
miR221	TCCAGCACCTAAGAAAATATGTGGC	CCCATGTACGTAATTTTAAACAACCTC
BFL1-1	GCAATGGGCACACCTGAAAG	GCCAAAGGGTAAAGCTCAAC
BFL1-2	ACAGTGGTTACCTCTTGGGAGA	CCTGTGTTGAAACTCATGTTGGTA
BFL1-TSS	TGGACCTGATCCAGGTTGTGGTA	TGCTCTCCACCAGGCAGAAG
BFL1-3	AGGAATTTGGCCTCCCAATCA	TTTCTCCAGCGACCATGAGTT
H3-YY1	GGAATAGAAGCGATGGGCTG	ATTCCTCTGGCCACTCATCA
RBS-YY1	AACTCTGGGTTATTGGGGCT	ACTACAAAGGTTCCTCCGCT
BFL1-4	AATCATAACTGCATGTGCCAAGTGC	CCTACACATGCCAAAGCTTCAGC
BFL1-5	GGGCAACAAGAGCGAAACTC	CTATGTCCTCGTCACACTTGGT
BFL1-6	ACAGGGGGTTTACGCTTGTC	TGCTAAACAAATGGGCTGGTTG
BFL1-7	CACCAATTCCGGACACAACAC	CTCAGCTCAGAAACAAGGTAAGG
BFL1-9	TTTTCCCCTCTCCCTGCTAGA	GGGGCCGCCATACTTTTGAG

Oligos used for chromatin conformation capture (3C); BFL-1 HindIII fragment		
	Forward	Modifications
TSS probe	TATAACCTGGGAGTTGAAGGGTTTTGCGGT	(5′FAM, 3′TAMRA)
TSS constant	TTAGAAACAGCACTTCCCTTTTTACTG	
−1	CAACTACCCACCTTACCTCTCTCC	
−3	CCGGATGAGATTACCATGGAGC	
−6	CAACGTATGCACTTTCTGCATGTTAT	
−9	GCAAATTAGGAGCATGATTGCCG	
−10	GGCTTCTGGTGAGGACTTCAG	
−11	CAGCAGTAGCAACCCGCTAG	
−12	CCCTTACCTTGTTTCTGAGCTGAG	
−13	GTCCCGTCTAGGCTTTCTCATTAC	
400-kb distance:		
+67	GATGAATTAGAAAGGTTAAAAAAC	
+68	ATTCAATATCTCAGATCTCTTTAGAAT	
+69	CTTAAAAATCATGCAGAGGCT	
+70	TAACTTGATTTAAAATCTTTGAAAGAATT	
+71	GATCTTCAGAATAATATAGTATTCTTTATCT	
sgRNA sequences (no PAM, 5′−3′)		
AAVS1	ggggccactagggacaggat	
BFL-1 TSS	GACACATGATGATACATGG	
Enh1 sg1	CTGGTGTGTTCTTTAGTTTC	
Enh1 sg2	GCTCCAGGAATACATACACG	
RBS sg1	ACCAAGCCTAGACCTCCCAG	
RBS sg2	ACAAGGCATGTAATGAACCA	
Enh2 sg1	CAAATGATTCACTCGCCTCG	
Enh2 sg2	ACCCATCTCATAAACTATAA	
Enh2 sg3	TGATAATACCAAGTGTGACG	
Enh2 sg4	CAAAATGGTATATCCGTACG	
ERE sg1	GATTTCCACCCTTCACAAAACC	
ERE sg2	CTGGAGTAGCTGGGACTACA	
PCR oligos for genomic DNA		
RBS (1,600 bp)	Forward: GATTTCCACCCTTCACAAAACC	Reverse: CTGTGTCATTGTGGGAAGTAGT

### Bioinformatics

Called peak files (.bed format) from tonsillar B-cell fraction and LCL (GM12878) ATAC-seq as well as ChIP-seq of EBNA-3 and histone modifications were prepared as described above or obtained from public repositories (11, 24, 27). Overlapping genomic regions across all ATAC-seq and ChIP-seq peaks were generated via multiple intersection using the *multiinter* function in bedtools with default parameters (28). The resulting intersection matrix was analyzed to identify combinatorial patterns of chromatin accessibility, epigenetic modifications, and EBNA binding among tonsil B-cell subsets and LCLs. Intersections across ATAC-seq peaks were visualized using the UpSetR package (v.1.4.0 [29]). ATAC and ChIP peaks matching user-defined criteria—such as those present in one tonsil fraction but not (!) another or those that intersected (∩) across data sets—were identified and used for *cis-*linked gene regulatory prediction (30) as described recently (31). Specific criteria were applied to identify genes linked to accessible chromatin regions associated with EBNA3A binding and enhancer histone modification signatures unique to tonsillar DZ and LZ B cells.

## RESULTS

### Epstein-Barr virus infection of primary human B cells *in vitro* phenocopies the germinal center reaction

**Fig 1:**
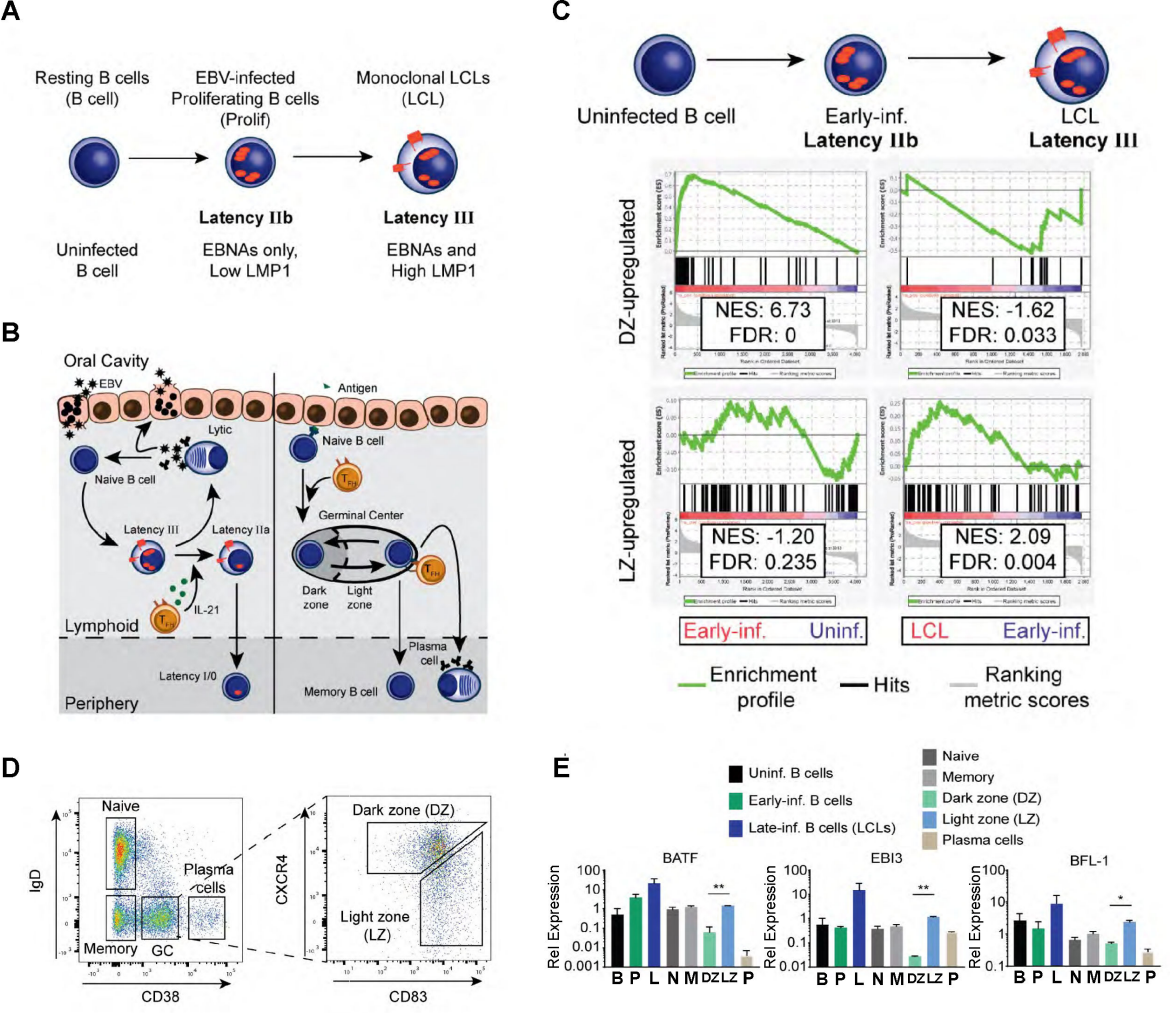
High BFL-1 expression typifies GC LZ B cells and EBV-immortalized LCLs. (**A**) Schematic of EBV infection of human B cells *in vitro* including transitions through latency IIb (EBNAs only) to latency III (EBNAs and LMPs). (**B**) Schematic comparing EBV infection *in vivo* with B-cell maturation in the oral cavity. Left, upon saliva transmission, EBV viral particles first infect an epithelial cell to amplify the initial viral load and increase the likelihood of infecting a naïve B cell in the lymphoid tissue. EBV infection activates the infected naïve B cell and stimulates it to proliferate through the expression of viral and host genes. First, EBV-infected B cells express the latency IIb gene expression program, in which all EBNAs are expressed. Then, the EBNAs activate the expression of the LMP, LMP1, and LMP2A, in latency III. IL-21 secretion by T follicular helper cells (T_FH_) in the lymphoid follicle induces the transition to latency IIa by silencing the expression of the EBNAs (33). Eventually, EBV-infected B cells attenuate viral gene expression in latency 0 but periodically express EBNA1 in latency I to maintain the viral genome in latently infected B cells. Plasma cell differentiation of EBV-infected B cells leads to lytic reactivation and production of infectious virion particles. Right, affinity maturation of B-cell antigen receptors is initiated when antigen encounter activates and stimulates naïve B cells to proliferate and form the GC reaction. In the GC DZ, hyperproliferating B cells undergo class-switch recombination and somatic hypermutation and transit to the GC LZ where they compete for antigen signaling and CD40 ligation from cognate T_FH_. Surviving B cells exit as plasma cells or as memory B cells. (**C**) Gene set enrichment analysis (GSEA) comparing differential gene expression between GC B cells and EBV-infected B cells *in vitro*. (**D**) Flow cytometry plots of sorting strategy of CD19^+^ B cell subsets from tonsillar lymphocytes. (**E**) qPCR comparing gene expression of BATF, EBI3, and BFL-1 from *in vitro* EBV-infected B cells and sorted tonsillar B cells. Data are representative of two experiments. Mean and SEM are plotted from two experiments and normalized to SETDB1 and uninfected B cells. Significance was determined by unpaired *t*-test between DZ and LZ B cells. *P < 0.05, **P < 0.01.

Previously, we found that EBV-infected B cells and uninfected, maturing B cells undergo dynamic, temporal regulation of apoptosis (8, 32). In particular, EBV-infected B cells and GC B cells upregulate strong dependence upon MCL-1 for survival, which led us to revisit the germinal center model of *in vivo* EBV infection. Originally proposed by David Thorley-Lawson, the GC model posits that EBV latent infection is established in maturing B cells undergoing the GC reaction. The primary difference between *in vivo* and *in vitro* EBV infection is that EBV infection of B cells *in vitro* generates immortalized LCLs that proliferate indefinitely in tissue culture (Fig. 1A). However, EBV infection *in vivo* is primarily established in quiescent memory B cells. Primary EBV infection takes place in the oral cavity, where viral particles transmitted through the saliva first infect oral epithelial cells (Fig. 1B). The infected epithelial cell amplifies the viral load, thereby increasing the likelihood of infecting naïve B cells present in the oral mucosa. EBV activates and stimulates infected B cells to proliferate through the expression of viral proteins, which include six EBNAs and two latent membrane proteins (LMPs). The specific patterns of viral gene expression are categorized as “latency” types and are temporally regulated to induce proliferation, survival, immune evasion, and re-infection (reviewed in reference 3).

From gene expression studies performed on tissue samples obtained from EBV^+^ lymphomas and infectious mononucleosis, it was observed that EBV latency programs mimic gene expression in antigen-activated, maturing B cells undergoing the GC reaction (Fig. 1B). The GC is spatially and functionally separated into two zones. In the DZ, antigen-activated naïve B cells undergo rapid hyperproliferation, class switch recombination, and somatic hypermutation of antigen receptors (14). In the LZ, B cells compete for survival signals in the form of CD40 and B-cell receptor (BCR) ligation from cognate T follicular helper cells. GC B cells possessing antigen receptors with subpar affinity are outcompeted and succumb to apoptosis (14). Surviving B cells exit as long-lived plasma cells and memory B cells or re-enter the DZ to undergo further affinity maturation (34, 35).

In our prior studies characterizing the early events after EBV infection, we identified several parallels between EBV infection and GC B cells. Similar to GC DZ B cells, early infected B cells undergo rapid hyperproliferation, activation of the DNA damage response, and upregulation of anti-apoptotic dependency upon myeloid cell leukemia protein 1 (MCL-1) (8). Combined with delayed upregulation of LMP1/NF-κB signaling in LCLs, we surmised that the early and late phases of *in vitro* EBV-infected B cells would be characterized by similar dynamic gene expression patterns in GC B cells. To determine a core set of genes that share similar dynamics of expression, we performed GSEA on differential gene expression between GC B cells and EBV-infected B cells. Using a pre-ranked list of DZ- or LZ-induced genes (14), we compared published microarray data that profiled gene expression changes in early and late infected B cells (4). This analysis revealed that genes upregulated in early infected B cells are enriched for targets induced in the DZ (Fig. 1C). Conversely, genes upregulated in LCLs are preferentially expressed in the LZ. This analysis is consistent with proliferation as a key defining trait for early infected B cells and cycling GC DZ B cells, and LMP1/CD40 (NF-κB) signaling is a hallmark for LCLs and GC LZ B cells.

Among the genes that made up the core GSEA enrichment set, EBI3, BATF, and BFL-1 (BCL2A1) were strongly upregulated in GC LZ B cells and LCLs. To confirm this finding, qPCR was performed on sorted tonsillar B cells (Fig. 1D) and EBV-infected B cells. LZ B cells exhibited significantly higher levels of EBI3, BATF, and BFL-1 mRNA compared to DZ B cells, as were LCLs compared to early infected B cells (Fig. 1E). In LCLs, we found that BFL-1 expression requires chromatin looping and epigenetic regulation by the viral nuclear protein EBNA3A (8). We, therefore sought to characterize the non-viral mechanisms underlying BFL-1 expression in GC LZ B cells.

### EBNA3A-dependent three-dimensional (3-D) enhancer looping supports YY1-regulated BFL-1 transcription in LCLs

**Fig 2:**
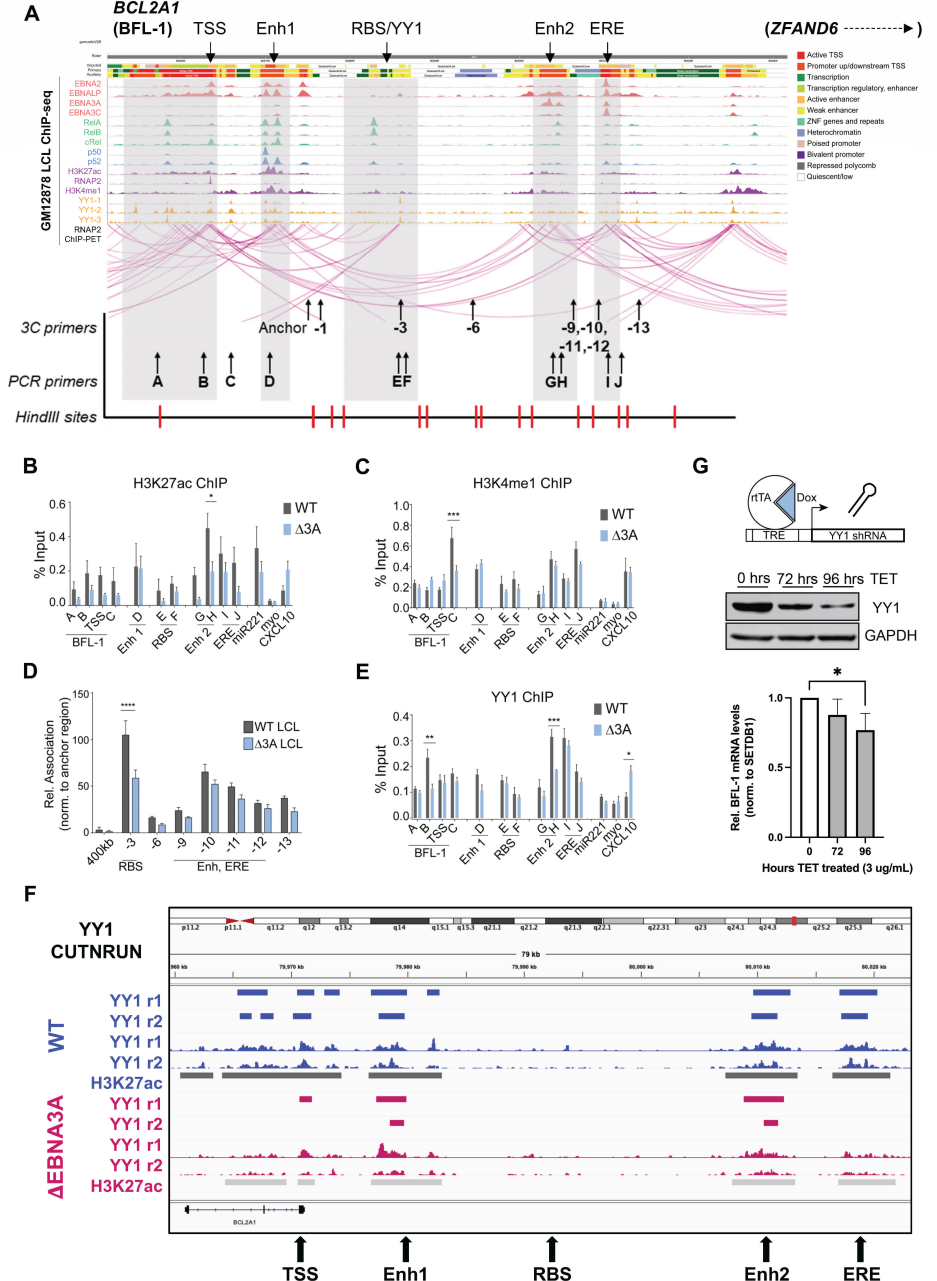
EBNA3A controls enhancer activity and 3-D chromatin interactions to promote YY1-mediated BFL-1 BFL-1 transcription in LCLs. (**A**) WashU Epigenome Browser screenshot of publicly available ChIP-seq and RNA Pol II ChIA-PET data sets performed on the GM12878 LCL, aligned to hg19 assembly, with labeled regions of interest (**i–iv**). ChromHMM annotations and tracks are included to show demarcated transcribed and active enhancer regions (41). (**B**) H327ac and (**C**) H3K4me1 ChIP-qPCR performed on WT and Δ3A LCLs. Regions corresponding to miR221 (EBNA3A-upregulated target), myoglobin (myo, not expressed in LCLs; negative control), and CXCL10 (EBNA3A-downregulated target) were also included. Enrichment calculated relative to input. Mean and SEM from three experiments are plotted. Significance determined by two-way analysis of variance (ANOVA). *P < 0.05, ***P < 0.001. (**D**) 3C-qPCR of interactions of HindIII fragments produced from WT and Δ3A EBV-immortalized LCLs. Interaction frequencies were normalized to those of the nearest neighbor HindIII fragment. Mean and SEM from three experiments are plotted. Significance determined by two-way ANOVA with Holm-Sidak’s multiple-comparison test. ****P < 0.0001. (**E**) YY1 ChIP-qPCR performed on WT and Δ3A LCLs. Enrichment calculated relative to input. Mean and SEM from three experiments are plotted. Significance determined by two-way ANOVA. *P < 0.05, **P < 0.01, ***P < 0.001. (**F**) YY1 CUT&RUN analysis at the BFL-1 locus for two independent WT (blue) and ΔEBNA3A (red) LCLs (R1 and R2). Auto-scaled sequencing tracks are shown along with bars indicating the SEACR called peaks. H3K27Ac CUT&RUN was also performed on one replicate each, and these peaks are shown as black and gray bars below the tracks indicating regulatory loci. (**G**) Tet-regulated YY1 shRNA was expressed in LCLs. Reduction of YY1 protein levels over 4-day time course (middle) and concomitant significant reduction of BFL-1 mRNA levels (bottom) (*n* = 3), *P < 0.05.

Previously, we found that BFL-1 transcription in LCLs was dependent upon EBNA3A (8). Specifically, the levels of active transcription machinery and chromatin looping to the BFL-1 TSS were dependent upon EBNA3A binding to distal enhancers. To further characterize the chromatin landscape at these distal sites, we integrated GM12878 LCL ChIP-seq data (13, 36–38) with annotations predicted by ChromHMM and LCL Chromatin Interaction Analysis by Paired-End Tag Sequencing (ChIA-PET) RNA Pol II data (Fig. 2A). ChromHMM is an automated computational system for learning, characterizing, and visualizing genome-wide maps of annotated chromatin states (39). RNA Pol II ChIA-PET data, which captures the long-range interactions of chromatin sites associated with transcription (40), revealed a high level of enhancer-promoter interactions between the BFL-1 TSS and several upstream regions. Based on these data, we decided to focus on the following regions in addition to the *BCL2A1* gene body: (i) enhancer 1 (Enh 1), a nearby enhancer bound by NF-κB transcription factors (37), EBNA2 (36) and EBNA-LP (38), and elevated levels of H3K27ac and H3K4me1; (ii) a RelA/B and YY1 binding region (RBS/YY1); (iii) enhancer 2 (Enh 2), a distal enhancer bound by RelA/B, H3K27ac and H3K4me1, and viral proteins EBNA-LP and EBNA3A (13); and (iv) the EBV regulatory element (ERE), a putative enhancer bound by all viral nuclear proteins.

We noted several chromatin regulatory features reliant on EBNA3A and suggestive of a role for YY1 in BFL-1 expression. First, we found that H3K27ac levels were elevated in WT LCLs compared to ΔEBNA3A LCLs, especially at Enh 2 (Fig. 2B). While H3K4me1 levels were mostly similar between WT and ΔEBNA3A LCLs, we observed an increase in H3K4me1 near the BFL-1 TSS in WT, but not ΔEBNA3A LCLs, which confirms our previous findings (Fig. 2C) (8). Reduced H3K27ac, but comparable levels of H3K4me1, at Enh 2 suggests that while this enhancer is adequately primed by bound chromatin regulators, its activity is dependent upon EBNA3A. Similarly, H3K27ac levels at the ERE did not change significantly in the absence of EBNA3A, suggesting that enhancer activity from this region is sufficiently maintained by the other EBNAs. Consistent with our prior work, EBNA3A was required for the 3-D loop architecture between the RBS and the BFL-1 TSS in LCLs (Fig. 2D). Given the importance of YY1 as a chromatin looping factor and its presence across the BFL-1 locus in LCLs, we examined the EBNA3A dependence in YY1 recruitment in LCLs. We observed that YY1 levels were reduced both distally at Enh 2 and within the BFL-1 gene body in EBNA3A-deficient LCLs (Fig. 2E). We subsequently validated these results using YY1 CUT&RUN where YY1 was significantly reduced at the ERE and within the BFL-1 gene body and moderately reduced at Enh2 and the BFL-1 TSS in the ΔEBNA3A relative to WT LCLs (Fig. 2F). Moreover, we used a tet-regulated YY1 shRNA depletion strategy to demonstrate that YY1 was important for BFL-1 expression in LCLs (Fig. 2G).

### Chromatin in GC B cells is significantly more accessible and has increased levels of histone marks that characterize regions of active transcription and enhancer activity

**Fig 3:**
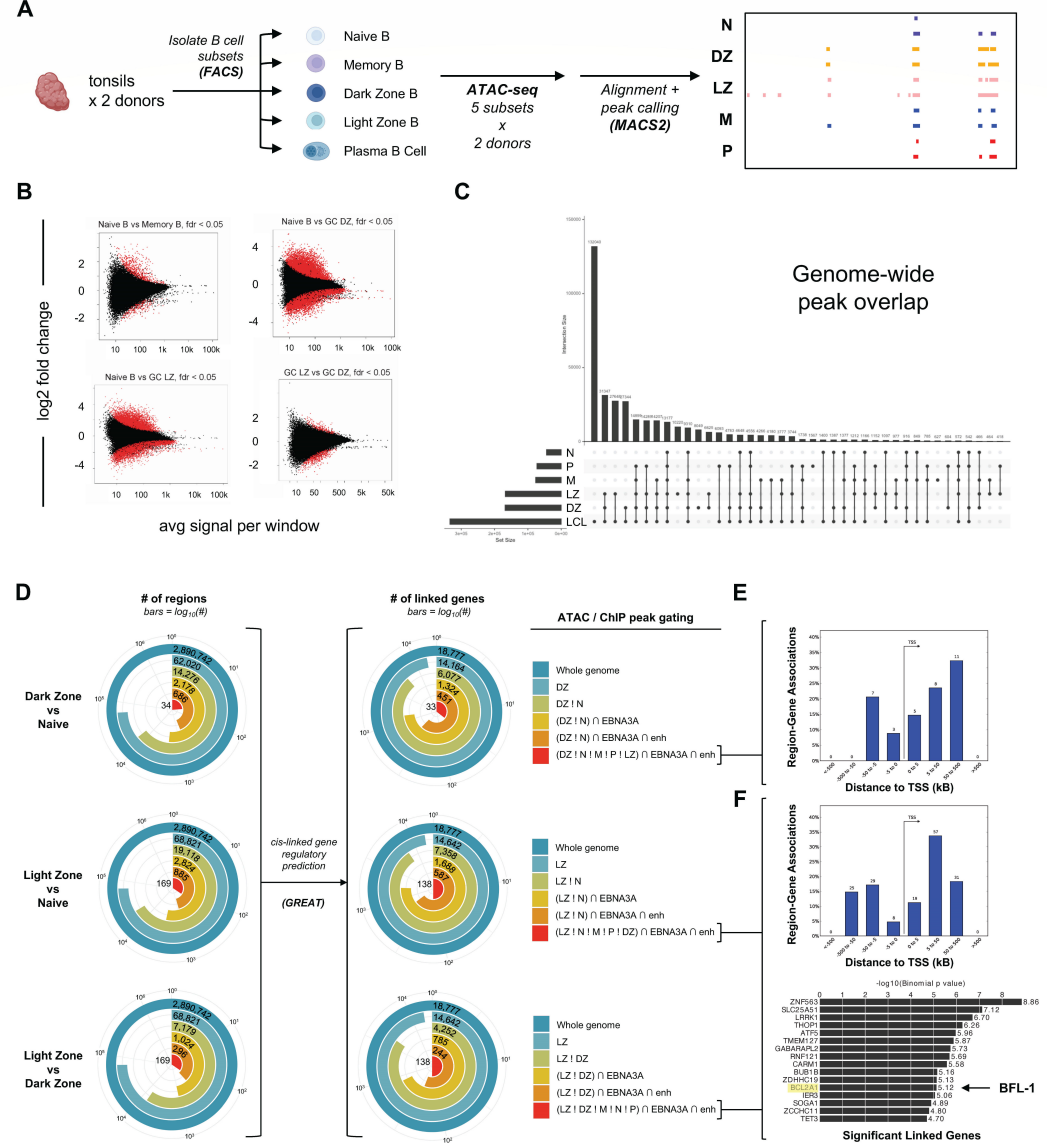
Identification of open chromatin regions in tonsillar B cells and prediction of genes linked to differentially accessible EBNA3A enhancer loci. (**A**) Schematic of ATAC-seq experiment indicating that data were generated from donor-matched CD19^+^ B cells sorted for naïve, memory, GC DZ, GC LZ, and plasma cells (PC). (**B**) MA plots (log_2_ fold change vs. mean average) showing differentially accessible regions between fractions of interest from tonsils. Red points indicate regions with a false discovery rate (FDR) <0.05. (**C**) Upset plot of genome-wide overlapping accessible chromatin regions among tonsillar B-cell subsets and LCLs. (**D**) Bullseye plots depicting the fraction of intervals with differential accessibility among tonsillar subsets, their co-incidence with EBNA3A and enhancer ChIP-seq signatures in LCLs, and cis-linked gene predictions. Logical gates were applied to filter ATAC peaks present in one subset but not in others (e.g., *LZ ! DZ*) and to intersect these differentially accessible loci against ChIP-seq peaks for EBNA3A and histone enhancer patterns (e.g., *∩ EBNA3A* and/or *∩ enh*). Regions matching given logical gating criteria were filtered to exclude any intervals shorter than 100 bases. Genes with predicted cis-regulatory linkages to intervals matching each set of gates were identified using GREAT (44). (**E**) Distribution of DZ-unique EBNA3A- and enhancer-associated gene-linked intervals relative to gene TSSs. This gate recipe [(*DZ ! LZ ! N ! M ! P) ∩ EBNA3A ∩ enh*] yielded no significant linked genes. (**F**) Distribution of LZ-unique EBNA3A- and enhancer-associated gene-linked intervals relative to gene TSSs. This gate recipe [(*LZ ! LZ ! N ! M ! P) ∩ EBNA3A ∩ enh*] yielded multiple statistically significant linked genes (binomial gene test FDR Q <0.05), including *BCL2A1* (BFL-1).

To characterize the mechanisms by which BFL-1 becomes upregulated in uninfected GC LZ B cells, we first used ATAC-seq to identify chromatin accessibility signatures in sorted tonsillar B-cell subsets (Fig. 3A). Overall, we found that chromatin is significantly more accessible in GC subsets compared to naïve B cells, which confirms previous studies that show that B-cell activation induces significant chromatin opening to expose biologically active regions (Fig. 3B) (42, 43). ATAC-seq also confirmed that naïve and memory B cells share highly similar chromatin landscapes. Notably, EBV-immortalized LCLs shared the highest genome-wide similarity in chromatin accessibility with GC B-cell states (DZ and LZ) rather than naïve, memory, or plasma cell subsets (Fig. 3C). Next, we evaluated genome-wide accessibility differences among naïve, DZ, and LZ subsets, specifically focusing on subset peak heterogeneity at potential EBNA3A-regulated sites. To do so, we generated a matrix of all intersecting peak ranges >100 bases across tonsil ATAC-seq samples and ChIP-seq data for EBNA3A, H3K27ac, H3K4me1, and H3K4me3 from the GM12878 LCL (13, 36–38). We applied increasingly stringent interval gating criteria to identify peaks present in one tonsillar subset but not in (! = “not in”; e.g., *LZ ! N* = peaks in LZ cells not found in naïve cells) as well as the number of subset-specific peaks co-incident with EBNA3A [*∩ = “*intersecting”; e.g., (*LZ ! N) ∩ EBNA3A*] and enhancer (“enh”) ChIP peaks [e.g., (*LZ ! N) ∩ EBNA3A ∩ enh*]. Genomic regions matching each gating recipe were used as inputs for GREAT (44) to predict genes potentially regulated in *cis* at these differentially accessible loci (Fig. 3D; Fig. S1). While we did not observe any statistically significant DZ-unique EBNA3A- and enhancer-linked genes (Fig. 3E), this informatic approach predicted a number of significant genes linked to EBNA3A enhancers, including *BCL2A1* (BFL-1), which were specific to LZ B-cell accessible chromatin sites (Fig. 3F). Based on the concordance between this predictive analysis and empirically measured gene expression, we sought to further validate and understand putative regulatory features associated with BFL-1 expression in GC LZ cells.

### 3-D enhancer looping and YY1 are associated with BFL-1 transcription in GC LZ B cells

**Fig 4:**
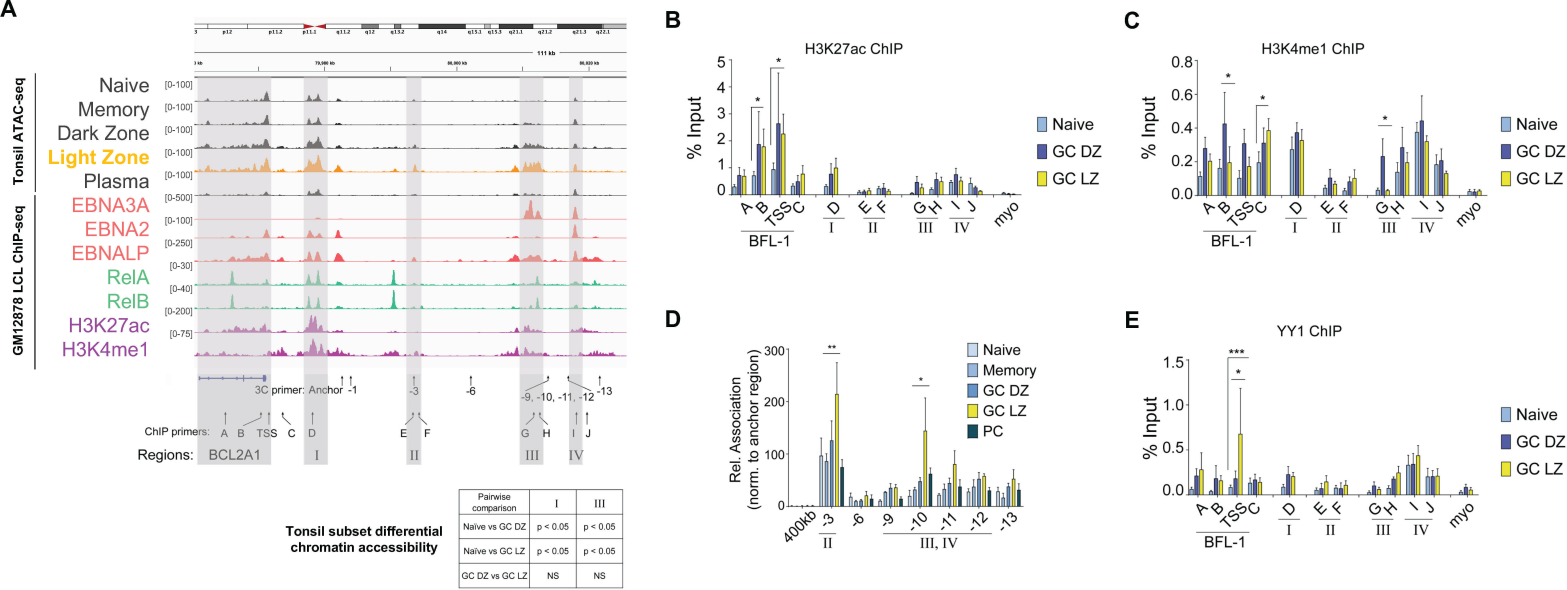
Increased enhancer activity and interactions to the BFL-1 TSS promote BFL-1 transcription in LZ GC B cells. (**A**) ATAC-seq profiles of sorted tonsillar B-cell subsets and select GM12878 ChIP-seq profiles. Light zone ATAC peaks are highlighted (gold) relative to other tonsil subsets (dark gray). ChIP-seq data are color coded by type (EBNAs in red; Rel TFs in teal; histone modifications in magenta). Relative positions of primers used for 3C-qPCR and ChIP-qPCR are included. Track heights are scaled to the visualized region and displayed in Integrated Genomics Viewer (IGV) using the hg38 assembly. Primers used for 3C-qPCR and ChIP-qPCR are listed below. Pairwise differential analysis on regions I and III performed on donor-matched replicates of ATAC-seq data from naïve, GC DZ, and GC LZ B cells. (**B**) H3K27ac and (**C**) H3K4me1 ChIP-qPCR performed on naïve, GC DZ, and GC LZ B-cell subsets. Enrichment calculated relative to input. Myoglobin (myo) was included as a negative control. Mean and SEM from three experiments are plotted. Significance determined by two-way ANOVA, multiple comparisons to the GC LZ population. *P < 0.05. (**D**) 3C-qPCR performed on sorted naïve, memory, GC DZ, GC LZ, and plasma cells from tonsillar CD19+ B cells. HindIII-produced fragments of chromatin were interrogated for interactions with the anchor primer near the BFL-1 TSS. Interaction frequencies were normalized to those of the nearest neighbor HindIII fragment, −1. Mean and SEM from three experiments are plotted. Significance determined by two-way ANOVA with Holm-Sidak’s multiple-comparison test. *P < 0.05, **P < 0.01. The HindIII fragment located 400 kb from the BFL-1 TSS served as a negative control. (**E**) YY1 ChIP-qPCR performed on naïve, GC DZ, and GC LZ B-cell subsets with enrichment normalized to input. Mean and SEM from three experiments are plotted. Significance determined by two-way ANOVA, multiple comparisons to the GC LZ population. *P < 0.05, ***P < 0.001.

Overall chromatin accessibility was not significantly different among B-cell subsets within the *BCL2A1* gene body. However, upstream regions I and III were found to be significantly more open in GC B cells compared to naïve B cells (Fig. 4A). Both GC subsets (DZ and LZ) shared similar chromatin openness, although LZ cells displayed enriched accessibility at region III (the EBNA3A-associated enhancer in LCLs) ~40 kb upstream of the *BCL2A1* TSS. While differential accessibility at this enhancer could contribute to the observed increase in BFL-1 expression in LZ versus DZ B cells, we reasoned that additional mechanisms may also be critical in regulating LZ-specific induction of BFL-1 mRNA.

We next sought to determine if chromatin regions were differentially activated at the *BCL2A1* locus among B-cell subsets. We found that H3K27ac levels were elevated in GC DZ and LZ subsets relative to naïve B cells and more so at the *BCL2A1* gene body than at the putative enhancer regions (Fig. 4B). B-cell subsets shared similar levels of H3K4me1 levels but were preferentially higher in GC DZ B cells near the BFL-1 TSS and enhancer III (Fig. 4C). To characterize the chromatin interactions at the BFL-1 locus in GC LZ B cells, we performed chromatin conformation capture analysis on sorted B-cell subsets. 3C-qPCR revealed cell type-specific chromatin structures upstream of the BFL-1 gene in which interactions between region I and regions II (−3) and III/IV (−10) were significantly higher in GC LZ B cells than in any other B-cell subset (Fig. 4D). Therefore, while GC DZ and LZ B cells share similar levels of chromatin accessibility, the formation of a specific three-dimensional chromatin architecture correlates with and likely facilitates BFL-1 upregulation in GC LZ B cells. The transcription factor YY1 is known to play a critical role in all stages of B-cell development, regulate the germinal center transcription program (45, 46), and generally facilitate enhancer-promoter interactions by dimerizing on chromatin (47, 48). Since we had found that EBNA3A-null LCLs had reduced levels of chromatin-bound YY1, we also performed YY1 ChIP-qPCR in sorted B-cell subsets. Among queried naïve, GC DZ, and LZ populations, YY1 occupies the chromatin landscape at similar levels but was uniquely and significantly higher at the BFL-1 TSS in GC LZ B cells (Fig. 4E). This suggests that YY1 is uniquely recruited in GC LZ B cells to facilitate the looping of activated enhancers to the BFL-1 TSS.

### BFL-1 transcription in LCLs results from combined activities of upstream genomic regions and enhancers

**Fig 5:**
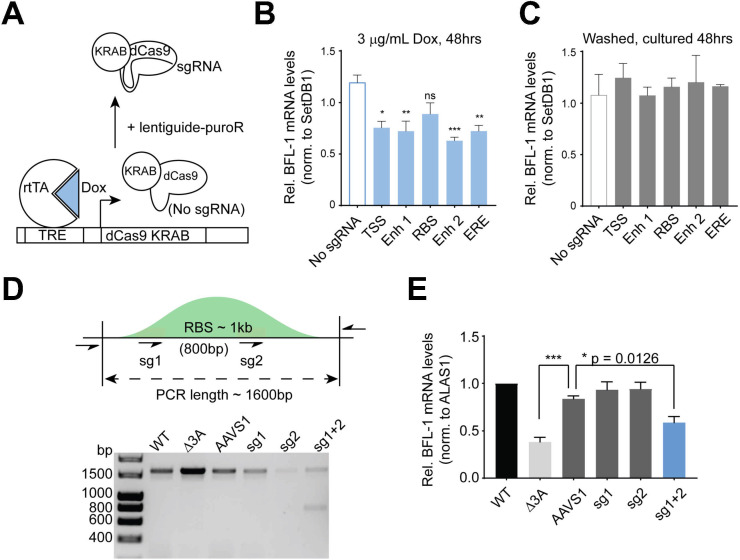
Upstream genomic regions are important for BFL-1 transcription, and BFL-1 protects against extrinsic apoptosis. (**A**) Schematic depicting TRE-dCas9-KRAB experimental system. Doxycycline (Dox) addition induces the expression of dCas9-KRAB but requires sgRNA expression from the lentiguide-puroR system to target the genomic DNA. (**B**) qPCR performed on LCLs transduced with TRE-dCas9-KRAB with or without sgRNAs targeting the BFL-1 TSS and upstream regions. Cells were treated with 3 mg/mL Dox for 48 hours. (**C**) Dox-treated cells are washed and then treated for 48 hours. Mean and SEM are plotted and normalized to untreated samples, with significance determined through one-way ANOVA with multiple comparisons to “No sgRNA” control. (**D**) DNA gel showing representative PCR confirming Cas9-mediated editing of the RBS region. PCR was performed on genomic DNA isolated from donor-matched WT and Δ3A LCLs and wild-type LCLs 5 days post-transfection with Cas9 coupled with sgRNAs targeting the AAVS1 site, sg1 and sg2 of the RBS, and combined sg1 and sg2. (**E**) qPCR performed on WT and Δ3A LCLs and WT LCLs transfected with Cas9 RNPs targeting the RBS. Results are from four separate transfections on two separate days, day 5 post-transfection. Significance by one-way ANOVA, multiple comparisons, and mean and SEM reported. *P < 0.05, ***P < 0.005.

To determine if upstream regions were important for BFL-1 transcription in LCLs, sgRNAs were targeted to the BFL-1 TSS, Enh 1, an RBS, Enh 2, and an ERE. These sgRNAs were co-expressed with dCas9-KRAB to repress transcription and enhancer activity (49). Because RNA Pol II ChIA-PET in LCLs showed high levels of interconnectivity between these regions (Fig. 2A), we surmised that constitutive inhibition of one enhancer could strengthen looping to the other enhancers to rescue BFL-1 expression, thereby masking the contribution of the targeted enhancer (50). Moreover, while BFL-1 expression is not crucial for LCL survival in the absence of extrinsic apoptotic signals (8), the BFL-1 enhancers also regulate the expression of ZFAND6, which is essential for LCL survival (51). We, therefore, utilized a tetracycline (TRE)-inducible dCas9-KRAB system to repress targeted enhancers (52, 53), such that doxycycline (Dox) treatment induced expression of dCas9-KRAB (Fig. 5A). Dox-induced repression of the TSS, Enh1, RBS, Enh 2, and ERE led to reduced BFL-1 mRNA levels compared to a non-targeting control (Fig. 5B), indicating that multiple regions contribute to BFL-1 expression in LCLs. Washing out doxycycline rescued BFL-1 knockdown, confirming drug-specific effects (Fig. 5C). The TSS, Enh 1, Enh 2, and ERE characterized by high H3K27ac peaks were consequently more sensitive to repression by dCas9-KRAB, which causes histone methylation and deacetylation (49). In contrast, the RBS had relatively low H3K27ac levels, so while dCas9-KRAB targeted at the RBS led to an observable decrease in BFL-1 mRNA levels, this did not achieve significance.

Nonetheless, because interactions between the RBS and the BFL-1 TSS fragments were so significantly enriched in WT LCLs, we hypothesized that the RBS was indeed important for BFL-1 transcription. We then used CRISPR/Cas9 to delete the RBS by using two sgRNAs that flanked the RelA and RelB ChIP-seq peaks (Fig. 5D). Using both guides together generated an approximately 800-bp deletion in the RBS (full length approximately 1,600 bp). Based on the relative intensities of the bands, approximately half of the alleles in transfected LCLs had major deletions in the RBS. This resulted in a significant reduction in BFL-1 mRNA levels (Fig. 5E). Transfecting either sgRNA individually led to limited editing, as determined by Sanger sequencing (Fig. S2), but no significant difference in BFL-1 expression. Thus, despite low H3K27ac levels, the RBS is an important NF-κB-regulated node for BFL-1 expression.

### BFL-1 promotes resistance against mitochondria-dependent extrinsic apoptosis

**Fig 6:**
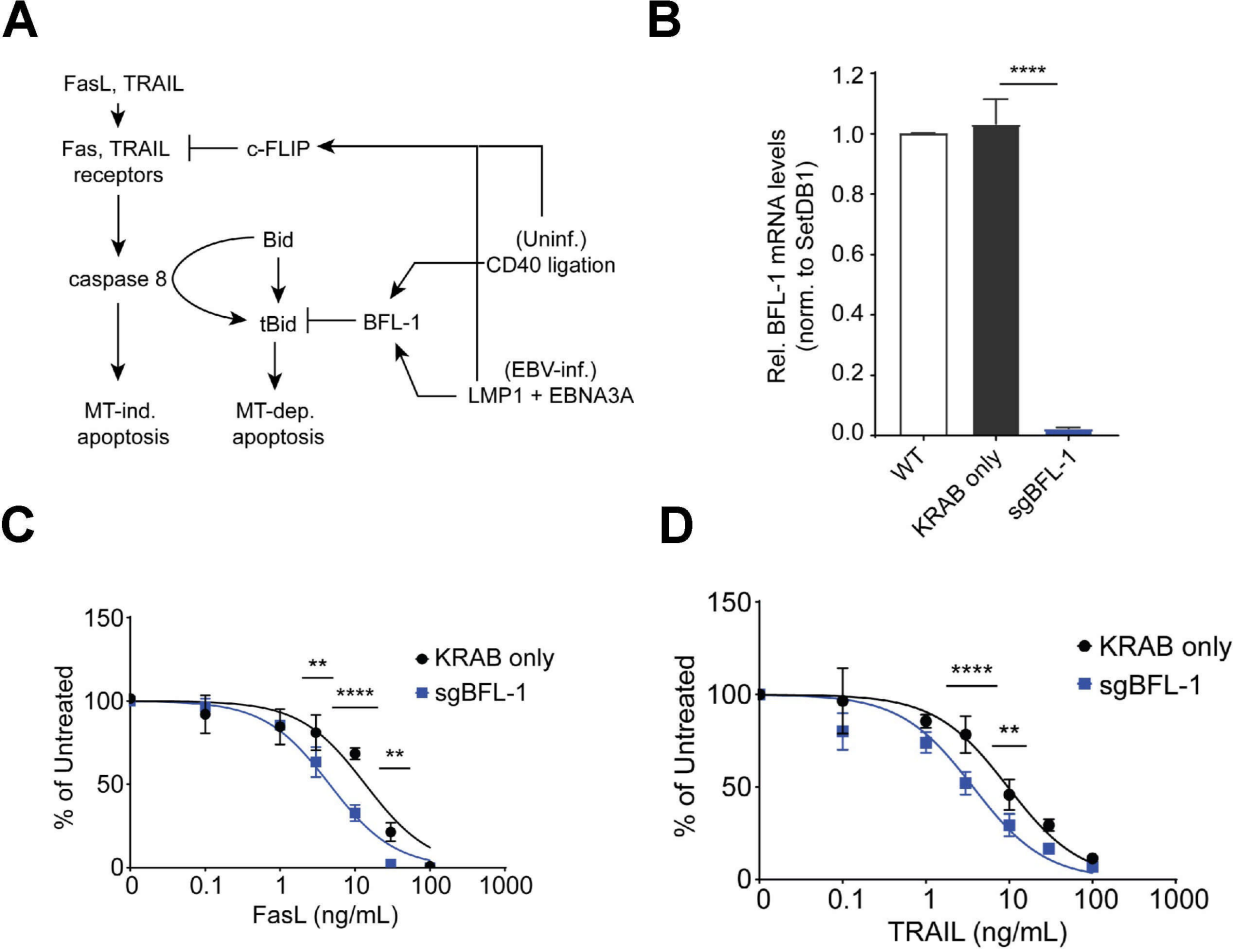
BFL-1 is important for protection against extrinsic apoptosis. (**A**) Schematic of FasL/TRAIL-mediated apoptosis of B cells in the germinal center light zone. NF-κB signaling in the form of CD40 ligation in uninfected B cells or LMP1 expression in EBV-infected B cells induces expression of c-FLIP, which inhibits FasL/TRAIL-induced extrinsic apoptosis. Downstream, BFL-1 upregulation protects against mitochondrial-dependent apoptosis induced by tBid. (**B**) qPCR of BFL-1 mRNA levels in WT LCLs and LCLs stably expressing dCas9-KRAB (only) and dCas9-KRAB targeted to the BFL-1 TSS (sgBFL-1). BFL-1 mRNA levels are normalized to SETDB1 and WT LCL. Mean and SEM from four experiments are reported. Significance determined by two-way ANOVA, multiple comparisons to sgBFL-1. ****P < 0.0001. (**C**) LCLs stably expressing dCas9-KRAB only (“KRAB only,” negative control) and dCas9-KRAB targeting the BFL-1 TSS (“sgBFL-1,” BFL-1 knockdown) were treated with TRAIL or (**D**) FasL for 3 days and then assayed for cell counts relative to untreated control. Mean and SEM from two replicates are reported. Significance determined by two-way ANOVA, comparing row cell means. **P < 0.01, ***P < 0.001, ****P < 0.0001.

NF-κB signaling, which is activated downstream of LMP1, maintains homeostasis in EBV-immortalized LCLs by promoting the expression of pro- and anti-apoptotic proteins. For example, NF-κB induces the expression of anti-apoptotic proteins BFL-1 and BCL-XL, which oppose pro-apoptotic sensitizers and effectors while also upregulating the expression of proteins involved in extrinsic apoptosis, such as Fas and TRAIL receptors and their cognate antigens (54). Upon ligand binding, Fas/TRAIL receptors recruit and oligomerize FADD proteins into a death-inducing signaling complex (DISC) that activates caspase 8, the initiator caspase in extrinsic apoptosis (Fig. 6A). Caspase 8 can directly activate executioner caspases 3/7 to induce mitochondria-independent apoptosis and can cleave the anti-apoptotic protein Bid into truncated Bid (tBid), a BH3-only peptide that induces intrinsic, mitochondria-dependent apoptosis. To prevent aberrant extrinsic apoptosis activation, NF-κB upregulates the expression of c-FLIP, which prevents DISC formation and was shown to be critical for protecting against tumor necrosis factor α (TNFα)-mediated extrinsic apoptosis in LCLs (51). This suggests that at steady state, LMP1 expression in LCLs leads to constitutive activation of extrinsic apoptosis that is sufficiently inhibited by upregulated levels of anti-apoptotic factors.

We hypothesized that BFL-1 upregulation in LCLs protects against mitochondrion-dependent extrinsic apoptosis. We generated LCLs stably expressing dCas9-KRAB constructs that targeted the BFL-1 TSS, which significantly ablated BFL-1 mRNA levels (Fig. 6B). While we cannot definitively exclude the possibility that steric effects of KRAB domain occupancy may affect expression, we attribute the loss of BFL-1 mRNA expression to the epigenetic repressive function of KRAB. BFL-1 knockdown LCLs were significantly more sensitive to increasing doses of FasL and TRAIL compared to negative control LCLs that expressed non-targeting dCas9-KRAB (Fig. 6C and D). Therefore, these experiments performed on LCLs support a role for BFL-1 in protecting against mitochondrial-dependent extrinsic apoptosis.

## DISCUSSION

Here, we have shown parallels between BFL-1 upregulation in EBV-immortalized LCLs and in uninfected GC LZ B cells. BFL-1 is one of several shared targets that are upregulated in EBV-infected B cells and GC LZ B cells. Previously, it was shown in LCLs that LMP1, EBNA2, and EBNA3A were important for BFL-1 transcription. LMP1-induced NF-κB signaling upregulates BFL-1 expression (55), EBNA2 activates BFL-1 transcription at the TSS (56), and EBNA3A is required for looping distal enhancers to the BFL-1 TSS (8). Loss of EBNA3A severely abrogates BFL-1 expression, which is still maintained at low levels due to EBNA2 and LMP1 activities. BFL-1 is, therefore, an important viral target in EBV infection, and its expression depends upon coordination among multiple viral proteins.

In GC B cells, chromatin at the BFL-1 locus and upstream regions becomes significantly more accessible and shares similar levels of H3K27ac. Increased chromatin interactions and YY1 binding at the BFL-1 TSS in GC LZ B cells promote BFL-1 transcription and a chromatin architecture that resembles that of WT LCLs. A direct relationship between EBNA3A and YY1 has not been shown previously, although both are important in mediating EBV superenhancer activity. However, as a polycomb group protein, YY1 may be recruited to EBNA3A-occupied sites to mediate chromatin looping and gene expression (57–60). In LCLs, functional interrogation of genomic regions using CRISPR and CRISPRi shows that BFL-1 expression is controlled by active enhancers and interacting domains. The similarities in chromatin regulation between LCLs and GC LZ B cells suggest that BFL-1 is an important target for both B-cell maturation and EBV infection.

The GC model posits that B cells infected by EBV *in vivo* transit through the GC reaction to gain access to the long-lived memory B-cell compartment. The data herein support this model by showing that an *in vitro* EBV infection of B cells phenocopies several important facets of GC B cell biology at the level of chromatin architecture, which complements our single-cell transcriptomic findings from early EBV infection and latently infected LCLs (61, 62). The GC reaction is functionally and spatially segregated into two zones, which are mimicked by the early and late phases of EBV infection. Both DZ B cells and early infected B cells undergo rapid hyperproliferation and induction of the DNA damage response, while LZ B cells and LCLs grown out from the late phase of EBV infection are characterized by CD40/BCR signaling (and subsequently arising differentiated cell fates [63]). Transcriptionally, early infected B cells are enriched for DZ-upregulated genes, and LCLs are enriched for LZ-upregulated genes, such as critical B-cell maturation factors like IRF4 and BATF that are essential for LCL survival (51). These observations indicate that many aspects of the GC reaction, such as dynamic regulation of transcription and chromatin regulation, are intrinsic to EBV infection. While not investigated here, applying similar techniques to study EBV infection of naïve tonsillar B cells may shed further light on the extent to which EBV engages normal GC B-cell programmed responses.

**Fig 7:**
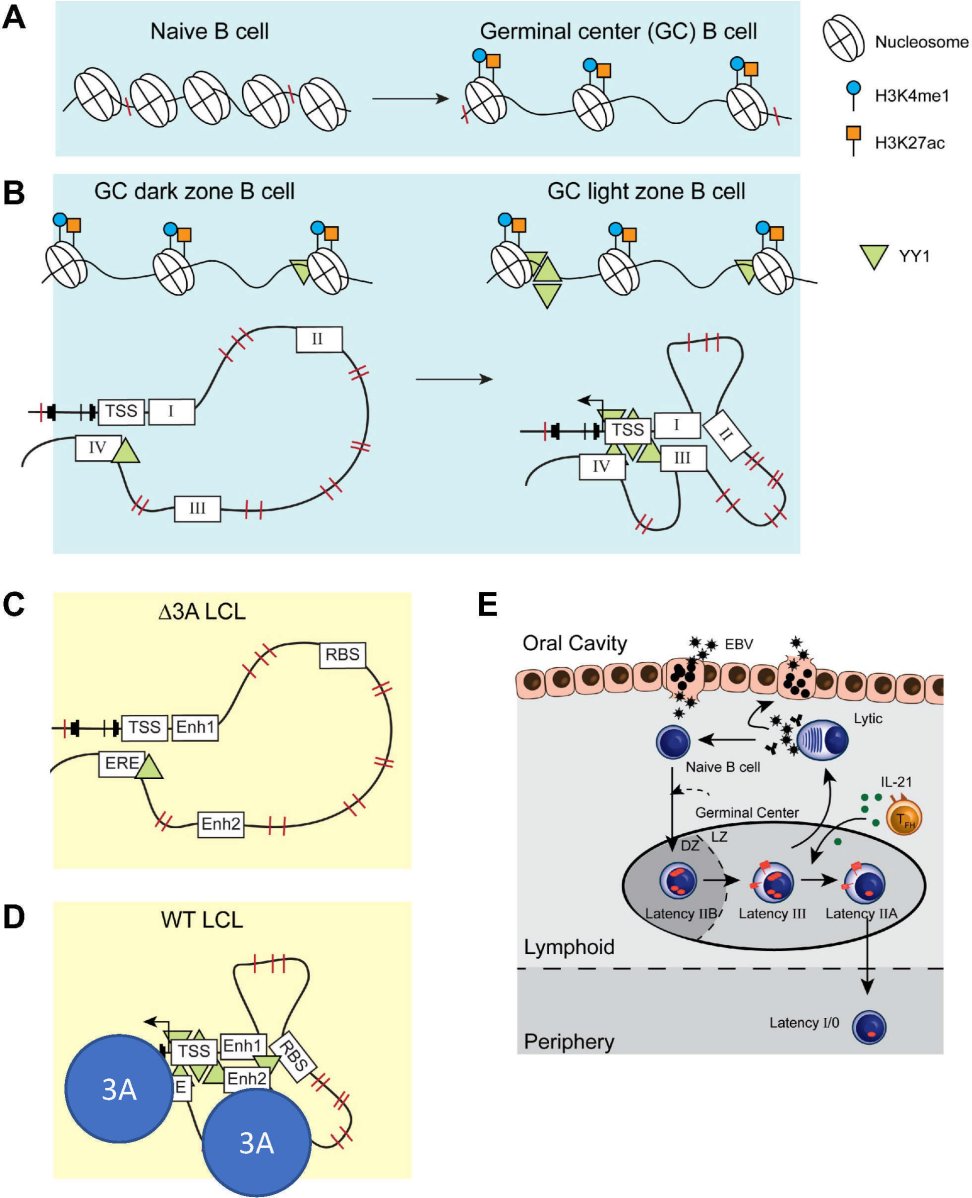
Illustrated schematic of stage-specific chromatin accessibility, activation, and looping in maturing B cells and EBV-infected B cells. (**A**) The transition from naïve to GC B cell is characterized by increased chromatin accessibility at *BCL2A1* and at enhancer regions. (**B**) At *BCL2A1,* increased YY1 deposition at the chromatin in GC LZ B cells facilitates the looping of activated and accessible enhancers in a specific chromatin architecture. This allows for LZ-specific upregulation of BFL-1 transcription. (**C**) EBNA3A-null LCLs have lower levels of chromatin-bound YY1 and lack the chromatin looping observed in (**D**) wild-type (WT) LCLs. Importantly, the chromatin architecture in WT LCLs strongly resembles that in GC LZ B cells. (**E**) A new, updated version of the GC model of EBV infection incorporates the gene expression similarities, anti-apoptotic dependencies, and chromatin regulation observed in *in vitro* models of EBV infection to those observed in maturing B cells.

We present an updated version of the GC model, based on this work and others from our laboratory (Fig. 7). In the GC DZ, EBV-infected B cells express the latency IIb program, which drives proliferation. Upon entering the GC LZ, EBV-infected B cells express LMP1 and LMP2A in the latency III program to promote survival. Originally, the GC model suggested that EBV-infected B cells expressed latency III and IIa in the GC, in which LMP1 and LMP2A promote proliferation and survival of the infected reservoir. However, LMP1 expression could be deleterious in that it induces potent cytotoxic T cell responses (64). In addition, EBV^+^ Burkitt lymphomas, which express high c-Myc but no LMP1, are relatively non-immunogenic (65). Therefore, the latency IIb program would permit rapid expansion of infected B cells with relatively low immunogenicity. This new GC model shows that the temporal regulation of viral gene expression observed in B cells infected *in vitro* is in accord with GC B-cell dynamics and biphasic NF-κB activity.

The substantial overlap of accessible chromatin between GC DZ B cells and LCLs reported here provides evidence for the infection-driven DZ mimicry component of this model. However, there are key distinctions between GC DZ B cells *in vivo* and EBV-induced DZ-like states. Previous studies have demonstrated that EBV downregulates CXCR4, a classic DZ biomarker, in early stages of infection (4, 66, 67). Infection also induces loss of another essential DZ regulator, FOXO1, in bulk sequencing studies (68). While these population-level effects are strong, data at the single-cell level indicate that subsets of EBV-infected cells in both early infection and LCLs retain or upregulate DZ mRNA for *CXCR4*, *FOXO1*, *AICDA*, *IL2RB*, *AURKC*, and *LMO2*. Moreover, actively proliferating cell subsets in early and latent infection contexts have *EZH2*, *TCF3*, and *BRCA1* expression signatures as observed in proliferating DZ B cells *in vivo* (61, 62, 69, 70). These apparent discrepancies in bulk vs single-cell data may be reconciled by Simpson’s paradox, wherein a globally observed trend in a heterogeneous population can be the inverse of the observed trend within subpopulations or phenotypes (71). Thus, both the nature of available experimental technique and the biological distinctions between normal GC DZ responses and their EBV-elicited analogs should be considered in the GC model of infection.

Likewise, there are important differences between GC LZ B cells and EBV-immortalized LCLs. For example, at the BFL-1 locus, H3K27ac was preferentially enriched at upstream enhancers in LCLs, whereas H3K27ac levels were higher at the BFL-1 gene than at enhancers in GC LZ B cells. This most likely reflects the fact that these viral-regulated enhancers control multiple targets. RNA pol II ChIA-PET shows that Enh 2 and the ERE are strongly linked to both BFL-1 and an upstream gene, *ZFAND6*, which is an LCL-essential gene (51). Also known as AWP1, ZFAND6 modulates NF-κB activity (72, 73), but a role for ZFAND6 in GC B cells has not been identified, despite the importance of NF-κB signaling in GC LZ B cells. It is possible that ZFAND6 may be more important for regulating sustained NF-κB signaling in LCLs rather than in transient settings such as the GC LZ. As for some DZ signature genes, the LZ biomarker *CD83* is downregulated upon infection in ensemble experiments but retained in EBV^+^ subsets co-expressing *BCL2A1* (BFL-1) and other GC LZ hallmarks (*CD80*, *CD86*, and *MYC*) (61, 62).

Comparison of bulk ATAC-seq data from tonsillar B-cell fractions and LCLs further highlights notable differences between EBV-immortalized cells and the GC LZ state. The extensive overlap of accessible chromatin intervals in LCLs with both LZ- and DZ-exclusive loci suggests that LCLs may reflect dysregulated GC B-cell biology or contain heterogeneous subpopulations that resemble distinct GC-like states. Recent single-cell RNA-seq studies from our laboratory provide evidence for such heterogeneity being established early during EBV infection and dynamically sustained after transformation (61, 62). In addition to the discrepancies described above, there are important discrepancies between *in vivo* GC B-cell phenotypes and their EBV-induced analogs in terms of BCL6 and c-Myc expression, both of which are required for the GC reaction. B cells infected with EBV *in vitro* strongly downregulate BCL6 expression (4, 74), suggesting perhaps that EBV infection is incompatible with the GC. However, EBV-infected B cells found *in vivo* can express BCL6, indicating that BCL6 expression is dependent upon environmental cues. While both early infected B cells and LCLs express c-Myc (2), c-Myc is rarely expressed in GC B cells but is nonetheless required for GC formation and plays a critical role in mediating chromatin changes in activated B cells (35, 43). Levels of c-Myc are upregulated among GC LZ B cells that re-enter the DZ to undergo further affinity maturation and their duration in the DZ depends upon the initial levels of c-Myc in the returning GC LZ B cell (75, 76). For an EBV-infected B cell, c-Myc is important for maintaining the latently infected state (77), but high c-Myc levels could lead to excessive retention in the GC and an increased risk of being detected and eliminated by T cells. EBV-infected B cells may overcome this challenge by restricting viral expression to latency IIa, which occurs in response to IL-21 secretion by T_FH_ (33). IL-21 silences EBNA2, the primary activator of c-Myc expression, and the EBNA3s, which are highly immunogenic. Thus, EBV-infected B cells are equipped to respond to appropriate cues and to overcome the barriers of the GC reaction—evidently via subversion rather than perfect recapitulation of normal GC dynamics.

The study of EBV infection *in vivo* has been challenging because primary EBV infection is often asymptomatic and, therefore, difficult to observe, and the frequency of infected B cells in asymptomatic cases is extremely low. As a result, much of what we know about *in vivo* infection has been informed by painstaking single-cell PCR experiments for viral transcripts and inferred from studies of EBV-associated diseases and tumors. The advent of improved chromatin technologies promises a better understanding of how EBV establishes latent infection in healthy individuals and how this becomes dysregulated in EBV-associated malignancies.

## Data Availability

ATAC-seq reads from sorted tonsillar B cell fractions used in this study are publicly available from the NIH Gene Expression Omnibus (GEO) via accession no. GSE159673. ATAC-seq reads from the GM12878 LCL used in this study were originally reported in reference 24 and are available via GEO accession no. GSE47753. Likewise, EBNA-3 ChIP-seq reads used herein were originally reported in reference 11 and are available via GEO accession no. GSE47629. Data and reagents used in this study will be made available upon reasonable request.

## SUPPLEMENTAL MATERIAL

Supplemental Figures mbio.02444-23-s0001.docx Fig. S1-S3.

ASM does not own the copyrights to Supplemental Material that may be linked to, or accessed through, an article. The authors have granted ASM a non-exclusive, world-wide license to publish the Supplemental Material files. Please contact the corresponding author directly for reuse.
