# Supplementary material for: Epstein-Barr virus induces germinal center light zone chromatin architecture and promotes survival through enhancer looping at the BCL2A1 locus
Source: mBio. 2023 Dec 7;15(1):e02444-23. doi: 10.1128/mbio.02444-23 (PMC10790771; doi:10.1128/mbio.02444-23)
Supplement: Supplemental Figures — Fig. S1-S3. [file mbio.02444-23-s0001.docx]

**Supplemental Figure 1. Predicted gene regulatory linkages at differentially accessible EBNA3A- and enhancer-associated loci in tonsillar subsets.**

**A)** Predicted genes with significant linkages to EBNA3A enhancer regions present in Dark Zone B cells and absent in Naïve B cells ((DZ ! N) ∩ EBNA3A ∩ enhancers). Enhancer regions were defined from GM12878 ChIP-seq data for H3K4me1, H3K4me3, and H3K27ac modifications. Main gene network (protein-protein interactions) and significant keyword and pathway terms (FDR < 0.05) for the predicted linked genes are shown.

**B)** Predicted genes linked to EBNA3 enhancer sites found in Light Zone B cells but not in Naïve B cells ((LZ ! N) ∩ EBNA3A ∩ enhancers). Data are presented as in **A)**, shown with significant GO process enrichment.


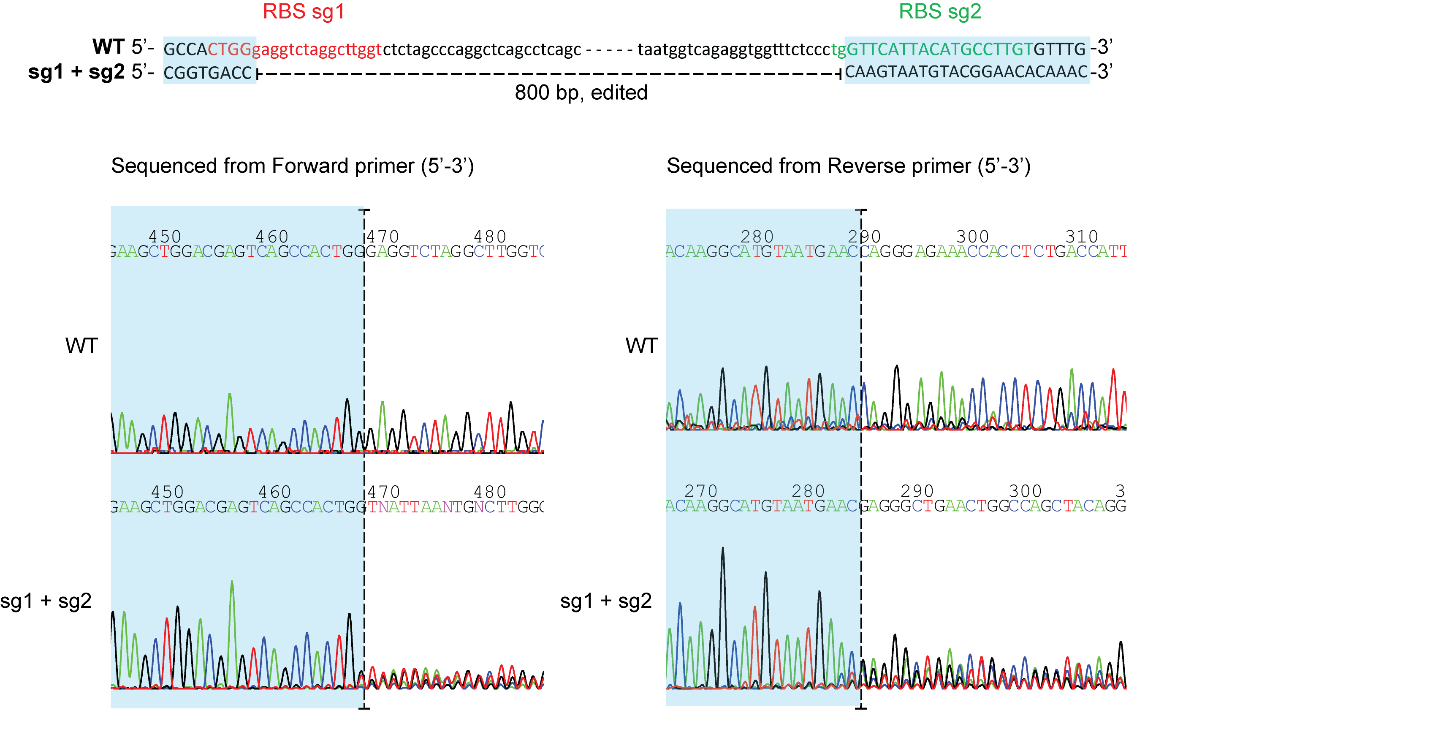


**Supplemental Figure 2**. **Bulk Sanger sequencing of edited RBS genomic region.** Due to major sequence alterations and deletions, editing efficiency was not quantifiable by Synthego ICE (Inference of CRISPR Edits) analysis. Trace files shown below. Sequences highlighted in blue are aligned.

**Supplemental Figure 3. Chromatin accessibility profiles in tonsillar B cell subsets and LCLs at key genes.** IGV tracks of ATAC-seq peaks at IRF4, MYC, BATF, BCL6, and BCL2A1 loci. Data are presented for each sorted tonsil B cell fraction from both donors and from publicly available ATAC-seq data for the GM12878 LCL (Buenrostro *et al*, Nature 2015 – GEO accession #GSE65360).
